# A Ras GTPase associated protein is involved in the phototropic and circadian photobiology responses in fungi

**DOI:** 10.1038/srep44790

**Published:** 2017-03-21

**Authors:** Silvia Polaino, José M. Villalobos-Escobedo, Viplendra P. S. Shakya, Alejandro Miralles-Durán, Suman Chaudhary, Catalina Sanz, Mahdi Shahriari, Eva M. Luque, Arturo P. Eslava, Luis M. Corrochano, Alfredo Herrera-Estrella, Alexander Idnurm

**Affiliations:** 1 Division of Cell Biology and Biophysics, School of Biological Sciences, University of Missouri-Kansas City, Kansas City, USA; 2 Laboratorio Nacional de Genómica para la Biodiversidad, CINVESTAV Sede Irapuato, Irapuato, Guanajuato, Mexico; 3 Departamento de Genética, Universidad de Sevilla, Sevilla, Spain; 4 Departamento de Microbiología y Genética, Universidad de Salamanca, Salamanca, Spain; 5 School of BioSciences, University of Melbourne, Australia

## Abstract

Light is an environmental signal perceived by most eukaryotic organisms and that can have major impacts on their growth and development. The MadC protein in the fungus *Phycomyces blakesleeanus* (Mucoromycotina) has been postulated to form part of the photosensory input for phototropism of the fruiting body sporangiophores, but the *madC* gene has remained unidentified since the 1960s when *madC* mutants were first isolated. In this study the *madC* gene was identified by positional cloning. All *madC* mutant strains contain loss-of-function point mutations within a gene predicted to encode a GTPase activating protein (GAP) for Ras. The *madC* gene complements the *Saccharomyces cerevisiae* Ras-GAP *ira1* mutant and the encoded MadC protein interacts with *P. blakesleeanus* Ras homologs in yeast two-hybrid assays, indicating that MadC is a regulator of Ras signaling. Deletion of the homolog in the filamentous ascomycete *Neurospora crassa* affects the circadian clock output, yielding a pattern of asexual conidiation similar to a *ras-1* mutant that is used in circadian studies in *N. crassa*. Thus, MadC is unlikely to be a photosensor, yet is a fundamental link in the photoresponses from blue light perceived by the conserved White Collar complex with Ras signaling in two distantly-related filamentous fungal species.

Eukaryotic life harnesses its energy from the sun, which cycles through a 24 hour period. Light represents a potent environmental signal available to organisms. In addition to sensing light, many species have also evolved circadian regulation to anticipate the time of day, using changes in light and temperature to set their clocks. The molecular mechanisms behind light-sensing and circadian clocks are being sought in ongoing molecular genetic investigations in a suite of organisms, including prokaryotes, animals, plants and fungi. Most species of this latter group respond to light. The fungi offer experimental benefits because they are non-photosynthetic and have haploid and unicellular growth stages.

Starting in the 1960s, two fungal species emerged as models for photobiology. Of these, the ascomycete *Neurospora crassa* became the predominant species for light and clock studies in the fungi. It was in *N. crassa* that the components to sense light, that is the White Collar (WC) complex, as well as to establish the circadian feedback loops, were first identified^1^,^2^. A key strain isolated in the 1960s was the *band (bd*) mutant, which provides a tight banding pattern of the asexual conidia approximately every 24 hours as part of the circadian output, whereas wild type strains have a diffuse pattern of conidiation^3^,^4^. The *bd* mutation was incorporated into the strains used in almost every subsequent circadian clock experiment. The mutation was only relatively recently identified by gene mapping approaches^5^. *Band* is an unusual allele of the gene encoding the small GTPase protein Ras^5^. The second fungus was *Phycomyces blakesleeanus*, a member of the Mucoromycotina. It became the research organism of choice of Max Delbrück, and was subjected to biophysical analyses, mutations and genetic analyses^6^,^7^. A series of mutants was isolated that were impaired in bending towards light (phototropism)^8^. However, the inability to transform *P. blakesleeanus* with foreign DNA prevented subsequent investigators from identifying the genes responsible^9^.

*P. blakesleeanus* produces asexual spore-forming structures, the sporangiophores, that are several cm long and about 0.1 mm wide and that bend towards blue light (Fig. 1A). The mutants with impaired phototropism led to the identification of ten different genes, named *mad* genes, defined by complementation tests and genetic linkage analyses^6^,^8^,^10^. The *mad* genes were subdivided into two classes, one involved in the photoreceptor function and the other in other tropic responses (known as the “stiff” mutants). Between two and five different photosensors were predicted for the species^11^. The *madA* and *madB* genes were identified as homologs of the *N. crassa wc-1* and *wc-2* genes, respectively, based on the hypothesis that these types of proteins would also regulate the blue light responses of *P. blakesleeanus*^12^,^13^. The phenotype in two *madI* mutants was recently identified as being caused by a situation in which the strains are heterokaryons, with a wild type copy and mutated copy of the *madA* gene^14^. The identity of the remaining seven *mad* genes could not be predicted based on the current knowledge of photobiology in the fungi.

Based on the *madC* phenotype by itself and through the analysis of strains combining *madC* mutations with mutations in other *mad* genes, MadC was predicted to be a photosensor or to interact with MadA or MadB^6^. The core domains for sensing light that are present in photoreceptor proteins are limited in number^15^ and few interacting components are known for the White collar complex in fungi. Hence, the discovery and characterization of *madC* could provide an advance in understanding photobiology, and was the impetus to identify the *madC* gene.

## Results

### Mapping-based identification of the *madC* gene

**Figure 1:**
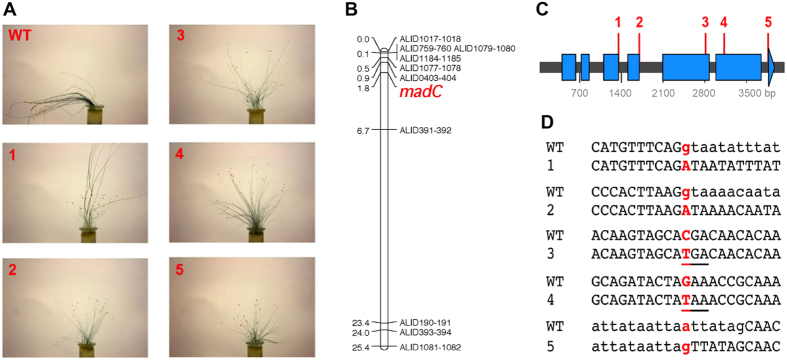
Identification of the *madC* gene of *P. blakesleeanus* by positional cloning. (**A**) Phenotypes of a wild type and five representative *madC* mutant strains. The effect of light, illuminated from the left side, on the sporangiophores of the wild type (WT) NRRL1555 and the allelic series of *madC* strains (1–5) grown in potato dextrose agar medium. (**B**) Segregation of the *madC* gene with molecular markers. Progeny (*n* = 93) from a cross between strains UBC21 × B2 were analyzed using PCR-RFLP markers. Marker names indicate the primers used for the amplification of the regions that are polymorphic between the two parents. Distances are in map units. (**C**) Position of mutations in the *madC* gene on a diagram of the gene, in which blue boxes indicate exons. 19 independent *madC* strains were examined: all bear a mutation along the gene. Numbers 1–5 indicate the alleles identified in the gene. The mutant strains have reduced phototropism and have allele 1: strains A202, A914, B2, B3, B4, S47, S193, S196 and S205; allele 2: strains C148, L1, L72 and S5; allele 3: strains C39, C54 and C93; allele 4: strains A491 and A492; allele 5: strain A905. (**D**) Nucleotide changes in the *madC* mutants compared to the wild type sequence. Lower-case letters are intron sequences. All the alleles would produce an impaired protein: alleles 1 and 2 affect the 5′ G of the intron, alleles 3 and 4 introduce premature stop codons (underlined), and allele 5 introduces a new 3′ splicing site.

To position the *madC* gene within the context of the genome sequence^14^, crosses were established between strain UBC21 (wild type, +) and two *madC* phototropism mutant strains, A914 and B2, of the (*−*) sex. Of the two crosses, B2 × UBC21 yielded a larger set of progeny, with 93 progeny isolated from 93 different zygospores: 35 were wild type and 58 were *madC* mutants. 56 PCR-RFLP markers were used on a subset of progeny to identify markers linked to the phototropism phenotype. The lowest recombination frequencies were found for markers on scaffolds located in linkage group IV^16^. Refined mapping with additional markers and using all 93 progeny indicated that the *madC* gene lies between RFLP marker amplified with primer combination ALID0403-ALID0404 in scaffold 33 and primer combination ALID0391-ALID0392 in scaffold 5 (Fig. 1B). The phototropic phenotype was less than 1 cM from the ALID0403-ALID0404 RFLP marker.

Taking the genetic linkage map of *P. blakesleeanus*^16^ and aligning to the genome sequence the markers that gave low recombination frequencies in the UBC21 × A914 and UBC21 × B2 crosses defined a small region in the genome, at the end of the scaffold 33, in which the *madC* gene could lie (Fig. 1B). The mutations in genes in *madC* strains were sought within this interval. Point mutations were identified in one gene (encoding protein id: 200804), initially in strain A914 and then in the two *madC* strains (A905 and B2) with available Illumina sequence data^14^. In comparison, 17 other *mad* mutants whose genome sequences are available did not bear mutations in this gene^14^.

### All *madC* strains have loss-of-function alleles in a gene encoding a Ras GTPase activating protein

By BLAST comparisons to the NCBI and *Saccharomyces cerevisiae* genome database, the protein impaired in *madC* strains is most similar to Ras GTPase activating proteins (GenBank KY522796). The candidate *madC* gene was sequenced from a larger collection of wild type and *madC* strains (Table S1). All *madC* strains carried mutations while no wild type or other strains did. Five different alleles were identified in the *madC* strains (Fig. 1C). All are predicted to produce non-functional proteins. Two mutations affect the most 5′ G nucleotides of introns, two introduce premature stop codons, and one introduces a new 3′ splicing site (Fig. 1D); the use of this new 3′ splice site for the *madC* allele in strain A905 was confirmed by sequencing the cDNA in that region. 19 of the *madC* mutants are independently-derived strains, suggesting hot spots for mutations, especially in intron splice sites as was observed previously with the *madB* mutants that all contain the identical mutation within an intron splice site^13^.

The mutations in the candidate *madC* gene were further linked to the impairment in phototropism by genetic segregation data using additional crosses (Data set S1). This is best illustrated in the UBC21 × A202 cross. The mutation, which in this case conveniently changes a restriction enzyme site, perfectly co-segregates with reduced phototropism, while recombination is seen between the phototropism phenotype and adjacent markers.

### MadC of *P. blakesleeanus* functions in Ras signaling

The *madC* gene encodes a putative Ras GTPase activating protein (GAP). Ras GAPs participate in the regulation of Ras activity by increasing the rate of hydrolysis of GTP to the inactive GDP-bound form. It is not possible to produce stably transgenic *P. blakesleeanus* strains^9^, limiting the possible experiments to perform in this fungus to further characterize MadC function. Instead, we used two approaches to provide evidence that the *P. blakesleeanus* MadC protein is a Ras GAP.

**Figure 2:**
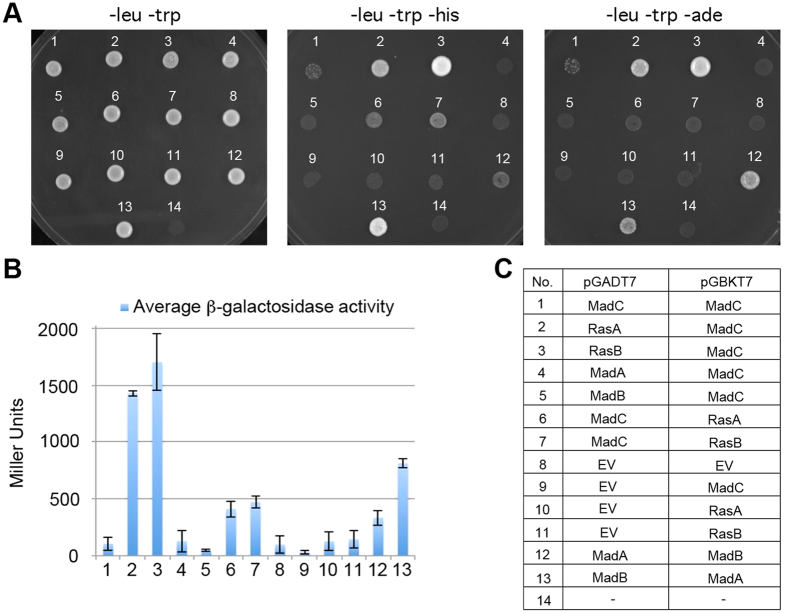
*P. blakesleeanus* MadC and Ras homologs physically interact in a yeast two-hybrid assay. *S. cerevisiae* strain PJ69-4A has the *ADE2, HIS3* and β-galactosidase genes under the control of galactose-inducible promoters. Physical interactions between proteins fused to the Gal4 activation domain (in plasmid pGADT7) and DNA binding domain (in plasmid pGBKT7) trigger the expression of these three reporters. (**A**) Growth of strains co-transformed with two plasmids on –leucine –tryptophan (–leu –trp) to maintain the plasmids, or on media to measure interactions (–histidine + 0.5 mM 3-amino triazole, or –adenine). (**B**) The average β-galactosidase activities of the strains, with standard errors from two biological replicates. (**C**) Arrangement of the cDNA clones in the plasmids with the Gal4 activation and DNA binding domains. EV; empty vector.

The first approach was the yeast two-hybrid interaction method to examine if MadC of *P. blakesleeanus* could physically interact with Ras homologs in its genome. Based on best reciprocal BLAST matches with the *S. cerevisiae* Ras1/Ras2 proteins, 10 candidate Ras homologs are present in *P. blakesleeanus*. Two genes encoding Ras homologous proteins were selected (id: 188551 and id: 177348), most closely related to the *S. cerevisiae* homologs and both of which are expressed, and their cDNAs isolated and cloned into yeast two-hybrid vectors along with the *madC* cDNA. Yeast strains expressing the *P. blakesleeanus* Ras homologs or MadC fused to the Gal4 domains grew on media without adenine or without histidine, and had higher β-galactosidase activity. These growth patterns and enzymatic activity indicate that MadC interacts physically with both RasA and RasB (Fig. 2). To explore further the relationship of MadC with other light-sensing components, the MadA and MadB homologs were cloned and expressed in the same yeast two-hybrid system. While MadA and MadB interacted in this system, as previously reported^13^, there was no evidence of interaction between MadC with either MadA or MadB.

**Figure 3:**
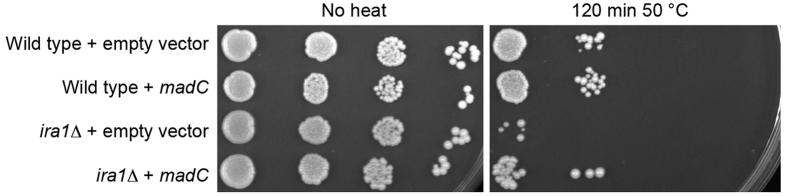
The *P. blakesleeanus madC* gene complements the heat shock sensitivity of the *S. cerevisiae ira1* mutant. The wild type strain of *S. cerevisiae* and an *ira1*Δ strain were transformed with an empty vector or with a vector expressing the *madC* gene. Ten-fold serial dilutions of the strains were plated on YPD medium. One plate was heat shocked at 50 °C for 120 min. The plates were then cultured 2 days at 30 °C.

The second approach was to explore whether MadC is a functional Ras GAP by cross-species complementation. *S. cerevisiae* encodes two Ras GAP homologs, Ira1 and Ira2, that exhibit different phenotypes in mutants depending on the parent strain background. These proteins, as the homologs of the NF1 protein of humans, have been extensively studied and the Ras GAP proteins have a high degree of functional conservation: for instance, human *NF1* can replace the functions of the two *IRA* genes in *S. cerevisiae*^17^. We deleted the *IRA1* homolog in *S. cerevisiae* strain W303, and then expressed the *P. blakesleeanus madC* cDNA clone under the control of the constitutive *ADH1* promoter in the *ira1*Δ strain. Deletion of *IRA1* in this *S. cerevisiae* genetic background renders the strains sensitive to exposure to heat shock^18^,^19^. Expression of the *P. blakesleeanus madC* gene in the *ira1*Δ strain restored in large part its heat shock sensitivity to wild type levels (Fig. 3). This result suggests that MadC is sufficiently conserved to function in regulating Ras function in this distant relative.

### Transcriptional changes caused by mutation of *madC*

A potential interaction between the light sensing White Collar complex and the Ras signaling system would be at the transcriptional level. The transcript abundances of *madA, madB* and *madC*, and the *cryA* gene encoding a cryptochrome as a photoregulated gene control^20^, were assessed in wild type and *mad* mutant strains grown under darkness or exposed to light, using quantitative reverse trancriptase PCR. No major differences were observed in the transcript levels of the *mad* genes in response to light (Fig. S1).

**Figure 4:**
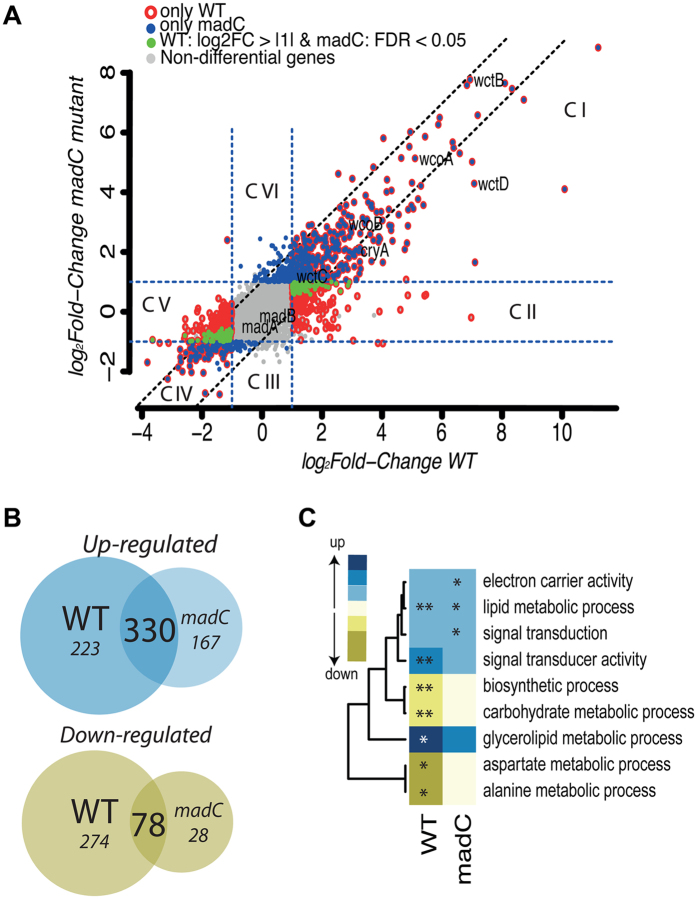
Comparison of the transcriptional response to light of wild type and *madC* strains. (**A**) Plot of gene transcript levels of the wild type vs *madC* strains by log_2_FC. Red circles represent differentially expressed genes in response to light for the wild type, and blue dots those differentially expressed in the *madC* strain (FDR <0.05; log_2_ fold-change ≥1). Grey dots represent genes that are not differentially expressed, and green dots are genes that do not pass the fold-change ≥|1| but have a significant change in the *madC* strain as determined by FDR. Diagonal lines (crossing the 0 of log_2_FC for both strains) serve as a reference to identify genes in the borderline of the cut-off criteria. (**B**) Venn diagrams showing the overlap of the repressed and induced genes in response to light between the wild type and *madC* strains. (**C**) Category enrichment in differentially expressed genes (*FDR <0.2; **FDR <0.05). Color intensity represents the percentage of genes belonging to each category and includes GO terms for Biological Process and Molecular Function, which contain less than 900 and more than 5 genes. The dendrogram highlights clusters based on percentages of genes contained in each gene ontology.

A global analysis of transcript changes was therefore undertaken using an RNA-sequencing approach. The transcriptional response of the wild type and *madC* mutant strains (Fig. 4A) indicated that overall the number of light regulated genes is reduced in the mutant, with 553 genes up-regulated and 352 genes down-regulated in the wild type (Supplemental Dataset 2), whereas there are 497 and 28 in the mutant (Supplemental Dataset 3), respectively. Based on their pattern of expression in the two strains, the set of light responsive genes can be separated into six clusters. Cluster I contains the 330 induced genes that are shared by the two strains (Fig. 4A,B). Notably, this cluster contains the paralogs of the genes *madA* and *madB* (i.e. *wcoA, wcoB, wctB, wctC, wctD*) and the *cryA* gene, representing all genes with a potential role in photoperception in *P. blakesleeanus*, and whose response to light is not affected by the mutation of the *madC* gene. Cluster IV encompasses 78 down-regulated genes that are shared between the two strains (Fig. 4A,B). Clusters III and VI contain the differentially expressed genes detected only in the *madC* mutant (167 up-regulated and 28 down-regulated), while clusters II and V contain those genes that appear to be responsive only in the wild type strain (Fig. 4A).

Interestingly, in general light responsive genes display a stronger response in the wild type than in the *madC* mutant (Fig. 4B), which is reflected in an important number of genes that pass the cut-off established [false discovery rate (FDR) <0.05; fold-change ≥2] in the wild type, and have the same tendency in the mutant but do not pass this filter. As highlighted by the intersection of the reference diagonal lines (crossing the 0 of log2FC) and the cut-off lines of log2FC, many of these genes are close to the cut-off that was established (Fig. 4A). This set of genes actually passes the FDR filter but does not fulfill the fold-change criteria (green dots in Fig. 4A). In this regard, we detected 94 up-regulated genes (FDR <0.05; fold-change ≥2) in the wild type that in the *madC* mutant show a FC ≥1.5. Similarly, among the down-regulated genes we found 133 fulfilling the cut-off criteria only in the wild type, and that in the *madC* strain show a FC ≤ −1.5. These comparisons allow us to conclude that most of the differentially expressed genes of the wild type are shared with the *madC* mutant, and that the corresponding gene is most likely involved in the modulation of the light response. It is, however, noteworthy that although both strains seem to activate genes related to signal transduction processes, in the mutant strain this GO category as well as that of lipid metabolic process is not significantly enriched (Fig. 4C; Table S2). Furthermore, there is no GO-term enrichment in the genes repressed in the mutant, whereas aspartate and alanine metabolic processes are enriched in the set of genes repressed in the wild type.

**Figure 5:**
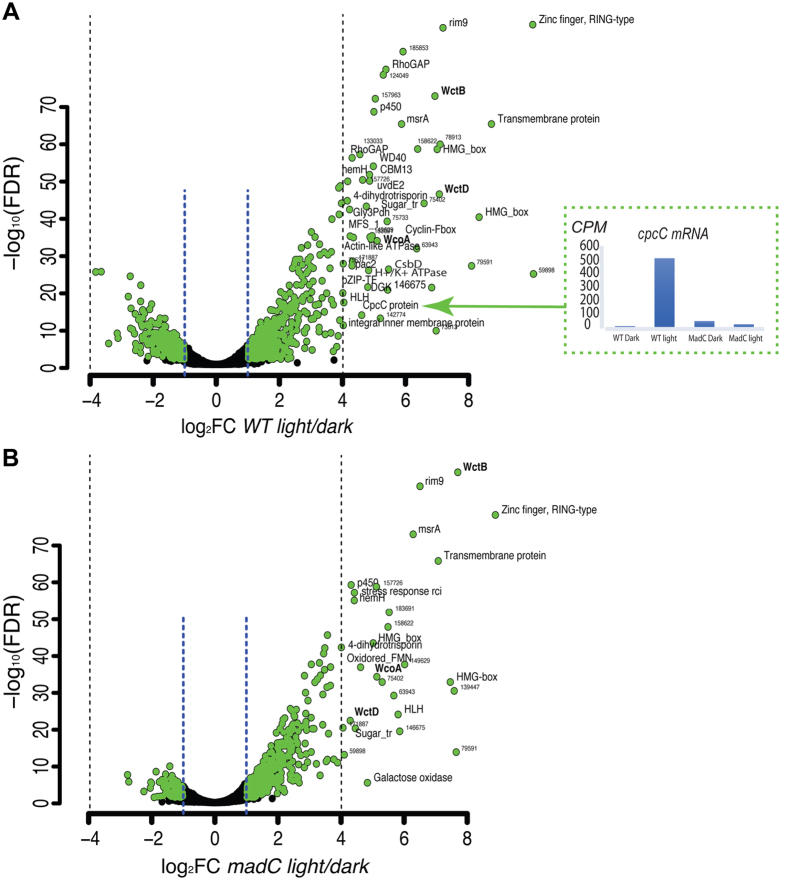
Gene expression profile in response to light of wild type and *madC* strains. Volcano plot of gene expression profile in response to light in the wild type (WT; **A**) and *madC* (**B**) strains, annotated as the proteins encoded by those genes. Plots of FDR versus log_2_ fold change for light/dark comparison in each strain. The two blue vertical lines in each chart mark two-fold change. Labels indicate representative genes with highly significant fold changes (log_2_FC >4). In A, the expression in counts per million (CPM) of the *cpcC* (homolog of clock-interacting protein CIPC) gene is shown.

The volcano plots in Fig. 5 show the differentially-expressed genes for both wild type (Fig. 5A) and *madC* (Fig. 5B) strains. These graphs illustrate a clear tendency for induction of gene expression in response to light rather than to repression in both strains. A set of very strongly induced genes (fold change higher than 16) called our attention since they could be guiding the main light response. In the wild type strain 50 genes have an expression level of this magnitude, and in the mutant only 30. Among these, 24 genes do not reach this level of expression in the *madC* strain. Within this group of transcripts affected by the *madC* mutation there are two Rho GTPases (id: 76783 & id: 152853). These proteins participate in the regulation of cytoskeletal rearrangements and vesicular traffic in eukaryotes and are part of the central elements that participate in the shift from isotropic to polarized growth in filamentous fungi, confirming that pathway signaling mediated by GTPases in the *madC* strain is affected^21^,^22^,^23^. *PAC2* (id: 6945) is another gene highly expressed in the wild-type strain and affected in *madC*; Pac2 participates in alpha-tubulin and beta-tubulin folding to be functional and null mutants of *pac2* are super-sensitive to benomyl, a microtubule depolymerizing drug^24^. These observations suggest that defects in vesicular transport may exist in the *madC* mutant. Another gene affected in the *madC* mutant encodes a diacylglycerol kinase (DGK, id: 175731) that catalyzes the conversion of diacylglycerol (DAG) to phosphatidic acid (PA), suggesting that phopholipid signaling in the light response is affected by the absence of *madC*. On the other hand, the *cpcC* gene (id: 121290) encoding the Clock-Interacting Protein (CIPC), a negative-feedback regulator of the mammalian circadian clock^25^, is strongly induced in the wild type strain with a fold-change greater than 19, whereas in the *madC* mutant this gene is not differentially expressed in response to light (see inset in Fig. 5A), and shows a significant decrease in expression even in the dark.

### The MadC homolog can impact photoresponses in other fungi

MadA and MadB (or WC-1 and WC-2, as named in *N. crassa*) are conserved in many fungi^26^,^27^. Likewise, homologs of MadC are found across the fungi and also in other eukaryotes (Figure S2). To address whether or not these homologs may also play roles in photobiology responses in other fungi, we tested their functions in a basidiomycete, *Cryptococcus neoformans*, and an ascomycete, *N. crassa*, which are both model species for photobiology research within their respective phyla.

**Figure 6:**
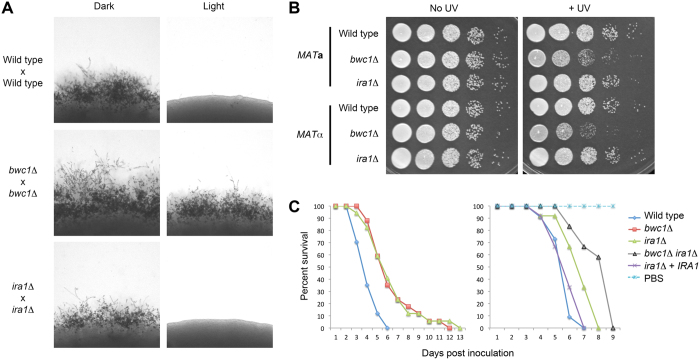
Phenotypes of the *C. neoformans ira1* mutant are not associated with light sensing. (**A**) Light represses mating in crosses between wild type or *ira1* mutant parents, but does not on the *bwc1* parents. Yeast cells were mixed on Murashige-Skoog medium and incubated in the dark or light. Mating is represented by the filaments emerging from the edges of the yeast colonies. (**B**) The *ira1* mutants have a wild type level of UV sensitivity in contrast to *bwc1* mutants. 10-fold serial dilutions of yeast cells were plated onto media, one plate was irradiated with 120 J/m^2^ UV, and strains grown 2 days to form colonies. (**C**) The *ira1* mutant has a reduction in virulence, with an additive effect when combined with the *bwc1* mutation. Wax moth larvae were inoculated for each strain, as indicated, in two independent experiments and survival monitored over time. Left graph *n* = 17; the wild type is more virulent than the two other strains (P < 0.001). Right graph *n* = 12; the differences between *ira1*Δ and *ira1*Δ + *IRA1* (P < 0.05) or *ira1*Δ and *bwc1*Δ *ira1*Δ (P < 0.05) are statistically significant.

The *madC* homolog (named *IRA1*; CNAG_06929, a single copy gene) in *C. neoformans* was deleted, and the strains tested for the three phenotypes associated with light-signaling in this fungus^28^. In contrast to the loss of the *white collar* homologs *BWC1* and *BWC2*, there was no effect of deleting the *madC* homolog on UV sensitivity or repression of mating by light (Fig. 6). A reduction in virulence was observed using an insect model of disease, like that seen for the *wc-1* mutant. A double *bwc1 ira1* mutant was isolated through genetic crosses, and its virulence tested. This strain showed an additional decrease in virulence, suggesting the actions of two independent pathways on virulence. Ira1 likely alters Ras signaling, known to be required for virulence in *C. neoformans*^29^,^30^. Thus, in this basidiomycete no clear role for the *madC* homolog in photobiology is evident.

**Figure 7:**
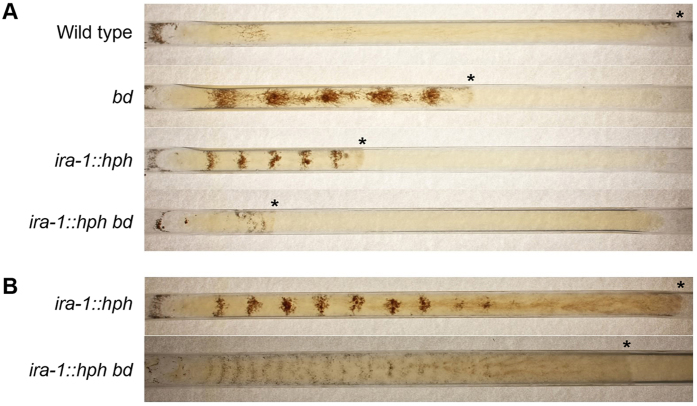
The *N. crassa* homolog of *madC* impacts circadian output. (**A**) The wild type strain, *bd* strain, the mutant in the *madC* homolog *ira-1::hph* and double *ira-1::hph bd* mutant were cultured in races tubes, growing from left to right, in light for 24 h at 25 °C and then transferred to constant darkness for 5 d at 25 °C. (**B**) Using similar culturing and initial illumination conditions as in (**A**), subsequently the *ira-1::hph* mutant was grown in darkness 14 days and the double *ira-1::hph bd* mutant grown in darkness for 28 days. Tubes are 16 mm in diameter. The * symbols indicate the positions of the growing fronts of the mycelia when photographed.

The function of the *madC* homolog in *N. crassa (ira-1*; NCU06122, a single copy gene) was examined. Previous studies have shown that Ras impacts the circadian output in *N. crassa*^5^. Homokaryon mutants without a copy of *ira-1* were isolated, by crossing a heterokaryon strain obtained from the *N. crassa* gene deletion project. Double *bd ira-1* mutants were isolated by performing additional crosses. The wild type, *ira-1::hph* mutant, *bd* strain, and double *ira-1::hph bd* mutant were grown in light and placed in races tubes in constant darkness. Strikingly, the *ira-1::hph* mutant produced rhythmic conidia approximately every 24 h like the *bd* mutant (Fig. 7A).

The *ira-1::hph* mutant grows slower than the *bd* mutant (Fig. 7A). Extending the incubation time to 14 days shows that the mutant continues to make bands of conidia up to about 10 days (Fig. 7B). The double *ira-1::hph bd* mutant exhibited additive effects, with slower growth than either the *ira-1::hph* or *bd* single mutants. Despite the slow growth, the double mutant was capable of forming bands in a circadian manner in complete darkness. The strength gradually diminished, but could still be faintly observed after 28 days (Fig. 7B). The reason why the RAS-1^bd^ protein isoform causes banding is unknown, as the *bd* mutant has little change in GDP/GTP ratio as compared to the wild type, but has a small decrease in the GDP exchange rate^5^. Belden *et al*. proposed that the RAS-1^bd^ protein may have altered interactions with guanine exchange factors; the additive effects of the double *ira-1::hph bd* mutant would be consistent with this hypothesis.

## Discussion

Two of the genes needed for phototropism in *P. blakesleeanus, madA* and *madB*, are the homologs of the *N. crassa wc-1* and *wc-2* genes, and were identified by predicting they were homologs of this conserved light-sensing complex^12^,^13^. The *madC* gene, encoding the other main photosensory component in the phototropism response, was unknown since the 1960s. In this study, we identify *madC* in *P. blakesleeanus* as a gene encoding a Ras GTPase activating protein.

The genomes of Mucoromycotina species often feature extensive gene duplication, either from whole genome and/or segmental duplication events^14^,^31^,^32^. For instance, the *madA* and *madB* genes of *P. blakesleeanus* and the homologous genes in other species in the Mucoromycotina are members of gene families with three or four genes, respectively^33^. The same situation is observed for homologs of the *madC* gene identified here, since *P. blakesleeanus* or related species have three or more *madC* homologs in their genomes (Figure S2). Considering the Ras pathway in *P. blakesleeanus*, the situation is even more complex, with ten Ras homologs in this species. However, the duplication of the Ras GAPs in *P. blakesleeanus* may have aided the discovery of this function in the phototropic response. This is because mutation of the homologs in other fungi can result in deleterious effects on growth. This includes the extreme phenotype of being essential for viability in some *S. cerevisiae* strain backgrounds^18^,^34^,^35^, and mutations causing growth or developmental defects in the basidiomycete *Schizophyllum commune*^36^ or ascomycetes *Aspergillus nidulans, Colletotrichum orbiculare* and *Schizosaccharomyces pombe*^37^,^38^,^39^. The *P. blakesleeanus madC* mutants have no defects in growth, development (e.g. mating or asexual sporulation) or other tropisms (gravity, avoidance response, photoinduction of sporangiophores or photoinduction of carotene synthesis) although a recent report suggests that one strain carrying a *madC* mutation may be hypersensitive to gravity^40^. The *madC* gene may have specialized its function after gene duplication to be specific to phototropism allowing the other Ras GAP proteins to perform their basic cellular roles.

The interactions between Ras signaling and photobiology are potentially widespread in eukaryotes. We tested the function of the homolog of the *P. blakesleeanus madC* gene in two divergent fungal species. While we did not see an effect on the responses to light in *C. neoformans*, this may reflect the possible life style of this species which is predominantly as a yeast^41^. In contrast, both the Ras GAP homolog of MadC and Ras itself are involved in the circadian output in *N. crassa*. The additive effect in *N. crassa* of the double mutant supports the hypothesis proposed by Belden *et al*.^5^ that the *bd* mutation impacts the interaction of Ras with guanine exchange factors: decreased growth rates in the *bd ira-1* double mutants are consistent with impaired growth seen for dominant negative and dominant active forms of Ras^5^.

Our transcriptome analysis of the *madC* mutant indicates that the function of this gene is not directly related to the transcriptional activation of the specific light responsive genes. However, congruently with the role in signaling processes of a GTPase, genes important for signal transduction are affected in response to the stimulus, since some of these genes are strongly induced in response to light and are no longer responsive in the mutant.

In *N. crassa*, the Rho-GTPase CDC-42 has been reported to participate in vesicular transport to the hyphal tip via the actin cytoskeleton and microtubules to carry out a thigmotropic response: this response depends on calcium-regulated supply of vesicles to the extreme hyphal apex via the actin cytoskeleton^42^. In the wild type strain of *P. blakesleeanus,* the phospholipase C (PLC; id: 121739) and diacylglycerol kinase DGK genes are induced, the latter is affected in *madC*. In both *N. crassa* and *C. albicans* PLC activity increases intracellular Ca^2+^ concentrations at the tip via inositol 1,4,5-triphosphate (IP_3_)-mediated calcium release from vesicles, and has been related to the thigmotropic response through a calcineurin-related mechanism^42^ and DGK is an essential enzyme in phosphatidic acid-dependent signaling pathways. The signaling cascade mediated by PLC and DGK is also involved in the phototropic movement of *Arabidopsis* chloroplasts and protection against high-intensity UV-B irradiation^43^,^44^. Thus, a light response network is described, which induces cytoskeletal modification and transport of vesicles to the tip of the sporangiophore, where there is an increase in calcium that can lead to phototropism in *P. blakesleeanus.*

One future direction is to explore if aspects of light sensing in *N. crassa* are altered in the *ira-1* mutant. Striking also is the role of the Ras GAP homolog in fruit fly *Drosophila melanogaster*, in which loss-of-function causes arrhythmic clock functions^45^. A potential mammalian link is the role of the Ras family member, Dexras1, in the light input into the circadian clock in mice^46^. Other studies continue to implicate an essential role for Ras signaling in circadian rhythms in animals^47^,^48^,^49^.

Interestingly, although in *P. blakesleeanus* a role for *madC* in circadian rhythms has not been established, the finding of the strongly affected expression level of the CIPC homolog correlates with the observed phenotype in the *ira-1* mutant in *N. crassa*. Thus, *madC* may have a highly conserved regulatory role in circadian rhythms.

In summary, we provide evidence that MadC is involved in Ras signaling in the life style of *P. blakesleeanus* to provide its ability to respond to light. It is remarkable that in two highly-diverged species of fungi, *P. blakesleeanus* and *N. crassa*, mutations that affect the same signaling pathway to alter photobiology were isolated in the 1960s, then remained unknown for over four decades. Future directions are to establish how MadC and Ras, or other Ras regulators interplay with the White Collar components to control the outputs of light signaling.

## Materials and Methods

### *P. blakesleeanus* strains and crosses

Details about the wild type and *mad* mutant *P. blakesleeanus* strains that were used are provided in Table S1. Crosses were established on V8 juice agar medium, and progeny isolated from zygospores two-four months later^16^.

### Mapping *madC*

DNA was isolated from the progeny from crosses between *mad* mutants [A914 and B2 (*−*)] with a wild type parent [UBC21 (+)] and used as the templates for using molecular markers developed previously to make a genetic linkage map of *P. blakesleeanus*^16^. Phototropism was scored by tropic reactions of sporangiophores growing on potato dextrose agar (PDA) medium in a homemade dark box with a glimmer window. The molecular marker and phototropism data were analyzed for linkage in JoinMap 4.0 software^50^.

### Yeast two hybrid analysis

The 5′ and 3′ ends of the *madC* gene were identified using rapid amplification of cDNA ends (GeneRacer kit, Invitrogen, Carlsbad, CA). A full-length cDNA clone was amplified with primers ALID1628-ALID1629 from reverse transcribed mRNA (SuperScript III First-Strand Synthesis System for RT-PCR, Invitrogen) and directly cloned using the EcoRI site into the pGADT7 and pGBKT7 plasmids. The *madA* cDNA was subcloned as an EcoRI fragment into the EcoRI sites of these two plasmids. The *madB* cDNA was subcloned as an NcoI-SalI fragment into the NcoI-XhoI sites of pGADT7 plasmid or NcoI-SalI sites of pGBKT7 plasmid. The *rasA* and *rasB* cDNAs were amplified with primers ALID1734-ALID1735 and ALID1736-ALID1737, respectively. The amplicons were recombined directly with the pGBKT7 plasmid in *S. cerevisiae*. The *rasA* and *rasB* cDNAs were amplified from these plasmids with primers ALID1829-ALID1830 and ALID1831-ALID1832, respectively; these amplicons were recombined directly with the pGADT7 plasmid in *S. cerevisiae*. The cDNA clones were sequenced to identify those without errors.

The plasmids and controls were co-transformed into *S. cerevisiae* strain PJ69-4A using the lithium acetate/PEG protocol^51^. Assessing the interactions of proteins followed the methods in James *et al*.^52^.

### Deletion of *S. cerevisiae IRA1* and complementation with the *P. blakesleeanus madC* gene

The *IRA1* gene was deleted in the *S. cerevisiae* strain W303 by replacing it with the selectable marker *KanMX6*, from the plasmid pFA6a-GFP (S65T)-KanMX6, amplified with primers SP98 and SP99. After transformation, the cells were plated onto YPD medium containing G-418. A gene replacement strain was identified and confirmed with different combination of primers, SP100 and KanB, KanC and SP101, SP100 and SP102, SP103 and SP101.

The *madC* cDNA was amplified with primers ALID1628 and ALID1629 and cloned into the EcoRI site of plasmid pTH19, which has expression from the constitutive *ADH1* promoter^53^. The W303 strain of *S. cerevisiae* and an *ira1::KanMX6* mutant were transformed with the control plasmid pTH19 and with the plasmid expressing the *madC* gene. The strains were inoculated in 5 ml YNB + glucose + leucine + tryptophan + adenine + histidine medium and grown overnight at 25 °C in shake cultures. Ten-fold serial dilutions of the strains were plated on YPD medium and exposed to 50 °C for up to 120 min, then allowed to grow two days at 30 °C.

### Disruption of the *madC* homologous gene (*IRA1*) in *C. neoformans* and characterization of mutant strains

The MadC homolog was identified by BLASTp. The corresponding gene was replaced with a construct conferring nourseothricin resistance by homologous recombination. The construct was made by overlap PCR using primers listed in Table S3, and transformed into strain KN99α by biolistic delivery, with subsequent selection on YPD medium containing nourseothricin (100 μg/ml). A *MAT***a** version was isolated by crossing this strain to congenic strain KN99**a**^54^, and a double *bwc1*Δ *ira1*Δ mutant was isolated by crossing. A complemented strain was generated by amplifying a wild type copy of *IRA1*, cloning it adjacent to a marker conferring G-418 resistance, and transforming this construct by *Agrobacterium*-mediated transformation in the *C. neoformans ira1*Δ mutant with selection on G-418 (100 μg/ml) and cefotaxime (200 μg/ml) for counter-selection of the bacteria.

The phenotypes of the strains were examined for inhibition of mating by light, UV sensitivity and virulence. Mating was tested by mixing strains of **a** and α mating types on Murashige-Skoog medium, and keeping one set under white light. UV sensitivity was tested by plating ten-fold serial dilutions onto YPD medium, and irradiating the plates with a UV transilluminator. The virulence of the strains were tested in the *Galleria mellonella* insect model^55^. Larvae were purchased from Vanderhorst Wholesale, Inc. (Saint Marys, OH). In brief, 1 × 10^5^ cells, from overnight cultures were washed and then resuspended in phosphate buffered saline (PBS), and then injected into the rear proleg of 12–17 larvae. Animal survival was monitored daily, and survival between groups compared using log-rank tests.

### Characterization of the *madC* homolog (*ira-1*) in *N. crassa*

*N. crassa* strains were obtained from the Fungal Genetics Stock Center (FGSC, http://www.fgsc.net)^56^. *N. crassa* genomic DNA was isolated using the ZR Fungal/Bacterial DNA kit (Zymo Research, Irvine, CA), and gene replacement validated by PCR. Strain manipulation and growth media preparation followed standard procedures and protocols^57^. Race tubes were inoculated, and incubated in constant light for 24 h at 25 °C before transfer to constant darkness at 25 °C (Advanced Intellus Environmental Controller Incubator, Percival Scientific, Perry, IA) for 5, 14 or 28 days, depending on the strain.

The strains *ira-1::hph bd* and *ira-1::hph* were generated by crossing the *ira-1::hph* strain FGSC 22825, from the *N. crassa* knockout project^58^, with the *bd* strain FGSC 1858. Mutations were confirmed by PCR and/or sequencing of amplicons. The phenotypes of the *ira-1::hph* mutants segregating from the cross were not like their parents; the *ira-1::hph* mutants (FGSC 22824 and FGSC 22825) are heterokaryons based on PCR analysis. The *ira-1::hph* homokaryotic strains were crossed with the wild type strain FGSC 4200, and strains of both mating type isolated, and deposited to the FGSC.

### Light induction, RNA isolation, quantitative RT-PCR and RNA-sequencing

10^4^ activated spores (48 °C, 15 min) of each *P. blakesleeanus* strain were grown at 22 °C in minimal medium. *P. blakesleeanus* dark-grown mycelia were exposed to blue light (2782.10 J/m^2^) at the age of 48 h for 30 min. After light exposure mycelia were collected and frozen in liquid nitrogen. Mycelia were disrupted by two 0.5-min pulses in a cell homogenizer (FastPrep-24, MP Biomedicals) with 1.5 g of zirconium beads (0.5 mm diameter) using the RNeasy Plant Mini Kit (Qiagen) following the manufacturer procedure. RNA samples were treated with DNase I prior to use in RT-PCR experiments. Total RNA was used for cDNA synthesis and amplification (primers in Table S3). Quantitative RT-PCR experiments were performed in a LightCycler 480 II (Roche, Madrid, Spain) using the One-Step SYBR PrimeScript RT-PCR kit (Takara Bio, Japan), 0.2 μl of each primer and 50 ng of RNA in 10 μl reactions. The reactions consisted of 5 min at 42 °C, followed by 10s at 95 °C, and then 40 cycles of DNA amplification (5 s at 95 °C and 20 s at 60 °C). The results for each gene were normalized to the corresponding results obtained with the actin gene (*act-1*) to correct for sampling errors. Then, the results obtained with each sample were normalized to the RNA sample from the corresponding mycelia after illumination.

Libraries for RNAseq were prepared with RNA from mycelia of the wild type and the A905 *madC* mutant strains using the TruSeq kit protocol (Illumina). Three biological replicates for each condition were sequenced using a HiSeq 2500 sequencer with the 1 × 100 format. 100-base-pair-long reads were obtained and mapped to the *P. blakesleeanus* genome V2 using bowtie^59^, searching for end-to-end hits with at most three mismatches. Alignment results were recorded in BAM format for further downstream analysis. Read counts per gene were calculated for each library using a shell script and collapsed into a table.

Differential expression analysis of gene expression in the wild type and *madC* strains in response to light was performed. Only genes with at least three reads per million were considered for differential expression analyses; this was done using the edgeR package^60^. Normalization of the data was performed using the Cox-Reid adjusted likelihood method. For determining differential expression between the comparisons, we used the Generalized Linear Model likelihood ratio test. False discovery rates (FDR) were calculated^61^ and genes with a FDR <0.05 and log_2_ fold-change ≥|1| were considered differentially expressed. Subsequently, we compared the differentially expressed genes of both strains in the plot of Figs 4A and 5, to highlight the genes with similar or different profiles between the two sets of genes.

A GO-term enrichment analysis was performed with the hypergeometric distribution method. GO terms with FDR ≤0.2 were considered significantly enriched in each comparison (Table S2). All RNAseq data have been deposited in the GEO database with accession number GSE93056.

## Additional Information

**How to cite this article:** Polaino, S. *et al*. A Ras GTPase associated protein is involved in the phototropic and circadian photobiology responses in fungi. *Sci. Rep.* **7**, 44790; doi: 10.1038/srep44790 (2017).

**Publisher's note:** Springer Nature remains neutral with regard to jurisdictional claims in published maps and institutional affiliations.

## Supplementary Material

Supplementary Material

Supplementary Dataset 1

Supplementary Dataset 2

Supplementary Dataset 3

## References

[b1] Lakin-Thomas P. L., Bell-Pedersen D. & Brody S. The genetics of circadian rhythms in *Neurospora*. Adv. Genet. 74, 55–103 (2011). 21924975 10.1016/B978-0-12-387690-4.00003-9 PMC5027897

[b2] Baker C. L., Loros J. J. & Dunlap J. C. The circadian clock of *Neurospora crassa*. FEMS Microbiol. Rev. 36, 95–110 (2012). 21707668 10.1111/j.1574-6976.2011.00288.x PMC3203324

[b3] Sargent M. L., Briggs W. R. & Woodward D. O. Circadian nature of a rhythm expressed by an invertaseless strain of *Neurospora crassa*. Plant Physiol. 41, 1343–1349 (1966). 5978549 10.1104/pp.41.8.1343 PMC550529

[b4] Sargent M. L. & Woodward D. O. Genetic determinants of circadian rhythmicity in Neurospora. J. Bacteriol. 97, 861–866 (1969). 5773032 10.1128/jb.97.2.861-866.1969 PMC249770

[b5] Belden W. J. . The *band* mutation in *Neurospora crassa* is a dominant allele of *ras-1* implicating RAS signaling in circadian output. Genes Dev. 21, 1494–1505 (2007). 17575051 10.1101/gad.1551707 PMC1891427

[b6] Cerdá-Olmedo E. *Phycomyces* and the biology of light and color. FEMS Microbiol. Rev. 25, 503–512 (2001). 11742688 10.1111/j.1574-6976.2001.tb00588.x

[b7] Cerdá-Olmedo E. & Lipson E. D. Phycomyces. (Cold Spring Harbor Laboratory Press, 1987).

[b8] Bergman K., Eslava A. P. & Cerdá-Olmedo E. Mutants of *Phycomyces* with abnormal phototropism. Mol. Gen. Genet. 123, 1–16 (1973). 4726375 10.1007/BF00282984

[b9] Obraztsova I. N., Prados N., Holzmann K., Avalos J. & Cerdá-Olmedo E. Genetic damage following introduction of DNA in Phycomyces. Fungal Genet. Biol. 41, 168–180 (2004). 14732263 10.1016/j.fgb.2003.09.007

[b10] Campuzano V., Galland P., Eslava A. P. & Alvarez M. I. Genetic characterization of two phototropism mutants of *Phycomyces* with defects in the genes *madI* and *madJ*. Curr. Genet. 27, 524–527 (1995). 7553936 10.1007/BF00314442

[b11] Galland P. & Lipson E. D. Blue-light reception in *Phycomyces* phototropism: evidence for two photosystems operating in low- and high-intensity ranges. Proc. Natl. Acad. Sci. USA 84, 104–108 (1987). 3540952 10.1073/pnas.84.1.104 PMC304150

[b12] Idnurm A. . The *Phycomyces madA* gene encodes a blue-light photoreceptor for phototropism and other light responses. Proc. Natl. Acad. Sci. USA 103, 4546–4551 (2006). 16537433 10.1073/pnas.0600633103 PMC1450208

[b13] Sanz C. . *Phycomyces* MADB interacts with MADA to form the primary photoreceptor complex for fungal phototropism. Proc. Natl. Acad. Sci. USA 106, 7095–7100 (2009). 19380729 10.1073/pnas.0900879106 PMC2678449

[b14] Corrochano L. M. . Expansion of signal transduction pathways in fungi by extensive genome duplication. Curr. Biol. 26, 1577–1584 (2016). 27238284 10.1016/j.cub.2016.04.038 PMC5089372

[b15] Briggs W. R. & Spudich J. L. Handbook of Photosensory Receptors. (Wiley-VCH, 2005).

[b16] Chaudhary S., Polaino S., Shakya V. P. S. & Idnurm A. A new genetic map for the zygomycete fungus *Phycomyces blakesleeanus*. PLoS One 8, e58931 (2013). 23516579 10.1371/journal.pone.0058931 PMC3597544

[b17] Xu G. F. . The catalytic domain of the neurofibromatosis type 1 gene product stimulates *ras* GTPase and complements *ira* mutants of S. cerevisiae. Cell 63, 835–841 (1990). 2121369 10.1016/0092-8674(90)90149-9

[b18] Tanaka K., Matsumoto K. & Toh-e A. *IRA1*, an inhibitory regulator of the RAS-cyclic AMP pathway in *Saccharomyces cerevisiae*. Mol. Cell. Biol. 9, 757–768 (1989). 2540426 10.1128/mcb.9.2.757-768.1989 PMC362653

[b19] Wang Y., Boguski M., Riggs M., Rodgers L. & Wigler M. *Sar1*, a gene from *Schizosaccharomyces pombe* encoding a protein that regulates ras1. Cell Regul. 2, 453–465 (1991). 1883874 10.1091/mbc.2.6.453 PMC361829

[b20] Tagua V. G. . Fungal cryptochrome with DNA repair activity reveals an early stage in cryptochrome evolution. Proc. Natl. Acad. Sci. USA 112, 15130–15135 (2015). 26578805 10.1073/pnas.1514637112 PMC4679004

[b21] Yorimitsu T., Sato K. & Takeuchi M. Molecular mechanisms of Sar/Arf GTPases in vesicular trafficking in yeast and plants. Front. Plant Sci. 5, 411 (2014). 25191334 10.3389/fpls.2014.00411 PMC4140167

[b22] Mucha E., Fricke I., Schaefer A., Wittinghofer A. & Berken A. Rho proteins of plants – functional cycle and regulation of cytoskeletal dynamics. Eur. J. Cell Biol. 90, 934–943 (2011). 21277045 10.1016/j.ejcb.2010.11.009

[b23] Riquelme M. & Martínez-Núñez L. Hyphal ontogeny in *Neurospora crassa*: a model organism for all seasons. F1000Res. 5, 2801 (2016). 27990280 10.12688/f1000research.9679.1 PMC5133687

[b24] Hoyt M. A., Macke J. P., Roberts B. T. & Geiser J. R. *Saccharomyces cerevisiae PAC2* functions with *CIN1, 2* and *4* in a pathway leading to normal microtubule stability. Genetics 146, 849–857 (1997). 9215891 10.1093/genetics/146.3.849 PMC1208055

[b25] Zhao W.-N. . CIPC is a mammalian circadian clock protein without invertebrate homologues. Nat. Cell Biol. 9, 268–275 (2007). 17310242 10.1038/ncb1539

[b26] Idnurm A., Verma S. & Corrochano L. M. A glimpse into the basis of vision in the kingdom Mycota. Fungal Genet. Biol. 47, 881–892 (2010). 20451644 10.1016/j.fgb.2010.04.009 PMC2950209

[b27] Rodriguez-Romero J., Hedtke M., Kastner C., Müller S. & Fischer R. Fungi, hidden in soil or up in the air: light makes a difference. Annu. Rev. Microbiol. 64, 585–610 (2010). 20533875 10.1146/annurev.micro.112408.134000

[b28] Idnurm A. & Heitman J. Light controls growth and development via a conserved pathway in the fungal kingdom. PLoS Biol. 3, 615–626 (2005). 10.1371/journal.pbio.0030095 PMC1064852 15760278

[b29] Alspaugh J. A., Cavallo L. M., Perfect J. R. & Heitman J. *RAS1* regulates filamentation, mating and growth at high temperature of *Cryptococcus neoformans*. Mol. Microbiol. 36, 352–365 (2000). 10792722 10.1046/j.1365-2958.2000.01852.x

[b30] Waugh M. S. . Ras1 and Ras2 contribute shared and unique roles in physiology and virulence of *Cryptococcus neoformans*. Microbiology 148, 191–201 (2002). 11782511 10.1099/00221287-148-1-191

[b31] Ma L.-J. . Genomic analysis of the basal lineage fungus *Rhizopus oryzae* reveals a whole-genome duplication. PLoS Genet. 5, e1000549 (2009). 19578406 10.1371/journal.pgen.1000549 PMC2699053

[b32] Schwartze V. U. . Gene expansion shapes genome architecture in the human pathogen *Lichtheimia corymbifera*: an evolutionary genomics analysis in the ancient terrestrial mucorales (Mucoromycotina). PLoS Genet. 10, e1004496 (2014). 25121733 10.1371/journal.pgen.1004496 PMC4133162

[b33] Corrochano L. M. & Garre V. Photobiology in the Zygomycota: multiple photoreceptor genes for complex responses to light. Fungal Genet. Biol. 47, 893–899 (2010). 20466063 10.1016/j.fgb.2010.04.007

[b34] Tanaka K. . *IRA2*, a second gene of *Saccharomyces cerevisiae* that encodes a protein with a domain homologous to mammalian *ras* GTPase-activating protein. Mol. Cell. Biol. 10, 4303–4313 (1990). 2164637 10.1128/mcb.10.8.4303-4313.1990 PMC360976

[b35] Harashima T., Anderson S., Yates J. R. 3rd & Heitman J. The kelch proteins Gpb1 and Gpb2 inhibit Ras activity via association with the yeast RasGAP neurofibromin homologs Ira1 and Ira2. Mol. Cell 22, 819–830 (2006). 16793550 10.1016/j.molcel.2006.05.011

[b36] Schubert D., Raudaskoski M., Knabe N. & Kothe E. Ras GTPase-activating protein Gap1 of the homobasidiomycete *Schizophyllum commune* regulates hyphal growth orientation and sexual development. Eukaryot. Cell 5, 683–695 (2006). 16607016 10.1128/EC.5.4.683-695.2006 PMC1459660

[b37] Harispe L., Portela C., Scazzocchio C., Peñalva M. A. & Gorfinkiel L. Ras GTPase-activating protein regulation of actin cytoskeleton and hyphal polarity in *Aspergillus nidulans*. Eukaryot. Cell 7, 141–153 (2008). 18039943 10.1128/EC.00346-07 PMC2224154

[b38] Harata K. & Kubo Y. Ras GTPase activating protein CoIra1 is involved in infection-related morphogenesis by regulating cAMP and MAPK signaling pathways through CoRas2 in *Colletotrichum orbiculare*. PLoS One 9, e109045 (2015). 10.1371/journal.pone.0109045 PMC4183519 25275394

[b39] Imai Y., Miyake S., Hughes D. A. & Yamamoto M. Identification of a GTPase-activating protein homolog in *Schizosaccharomyces pombe*. Mol. Cell. Biol. 11, 3088–3094 (1991). 2038319 10.1128/mcb.11.6.3088-3094.1991 PMC360150

[b40] Göttig M. & Galland P. Gravitropism in *Phycomyces*: violation of the so-called resultant law - evidence for two response components. Plant Biol. 16 Suppl 1, 158–166 (2014). 24373014 10.1111/plb.12112

[b41] Lin X. *Cryptococcus neoformans*: morphogenesis, infection, and evolution. Infect. Genet. Evol. 9, 401–416 (2009). 19460306 10.1016/j.meegid.2009.01.013

[b42] Stephenson K. S., Gow N. A. R., Davidson F. A. & Gadd G. M. Regulation of vectorial supply of vesicles to the hyphal tip determines thigmotropism in *Neurospora crassa*. Fungal Biol. 118, 287–294 (2014). 24607352 10.1016/j.funbio.2013.12.007

[b43] Aggarwal C., Łabuz J. & Gabryś H. Phosphoinositides play differential roles in regulating phototropin1- and phototropin2-mediated chloroplast movements in *Arabidopsis*. PLoS One 8, e55393 (2013). 23405144 10.1371/journal.pone.0055393 PMC3566141

[b44] Yoon M. Y. . Transcriptomic profiling of soybean in response to high-intensity UV-B irradiation reveals stress defense signaling. Front. Plant Sci. 7, 1917 (2016). 10.3389/fpls.2016.01917 PMC5165247 28066473

[b45] Williams J. A., Su H. S., Bernards A., Field J. & Sehgal A. A circadian output in *Drosophila* mediated by *Neurofibromatosis-1* and Ras/MAPK. Science 293, 2251–2256 (2001). 11567138 10.1126/science.1063097

[b46] Cheng H.-Y. M. . The molecular gatekeeper Dexras1 sculpts the photic responsiveness of the mammalian circadian clock. J. Neurosci. 26, 12984–12995 (2006). 17167088 10.1523/JNEUROSCI.4253-06.2006 PMC6674968

[b47] Weber F., Hung H.-C., Maurer C. & Kay S. A. Second messenger and Ras/MAPK signalling pathways regulate CLOCK/CYCLE-dependent transcription. J. Neurochem. 98, 248–257 (2006). 16805811 10.1111/j.1471-4159.2006.03865.x

[b48] Relógio A. . Ras-mediated deregulation of the circadian clock in cancer. PLoS Genet. 10, e1004338 (2014). 24875049 10.1371/journal.pgen.1004338 PMC4038477

[b49] Serchov T. . Ras activity oscillates in the mouse suprachiasmatic nucleus and modulates circadian clock dynamics. Mol. Neurobiol. 53, 1843–1855, doi: 10.1007/s12035-015-9135-0 (2016). 25762011

[b50] Ooijen J. W. V. & Voorrips R. E. JoinMap 3.0: Software for the Calculation of Genetic Linkage Maps. (Plant Research International, 2001).

[b51] Ito H., Fukuda Y., Murata K. & Kimura A. Transformation of intact yeast cells treated with alkali cations. J. Bacteriol. 153, 163–168 (1983). 6336730 10.1128/jb.153.1.163-168.1983 PMC217353

[b52] James P., Halladay J. & Craig E. A. Genomic libraries and a host strain designed for highly efficient two-hybrid selection in yeast. Genetics 144, 1425–1436 (1996). 8978031 10.1093/genetics/144.4.1425 PMC1207695

[b53] Harashima T. & Heitman J. Gα subunit Gpa2 recruits kelch repeat subunits that inhibit receptor-G protein coupling during cAMP-induced dimorphic transitions in *Saccharomyces cerevisiae*. Mol. Biol. Cell 16, 4557–4571 (2005). 16030250 10.1091/mbc.E05-05-0403 PMC1237064

[b54] Nielsen K. . Sexual cycle of *Cryptococcus neoformans* var. *grubii* and virulence of congenic **a** and α isolates. Infect. Immun. 71, 4831–4841 (2003). 12933823 10.1128/IAI.71.9.4831-4841.2003 PMC187335

[b55] Mylonakis E. . *Galleria mellonella* as a model system to study *Cryptococcus neoformans* pathogenesis. Infect. Immun. 73, 3842–3850 (2005). 15972469 10.1128/IAI.73.7.3842-3850.2005 PMC1168598

[b56] McCluskey K., Wiest A. & Plamann M. The Fungal Genetics Stock Center: a repository for 50 years of fungal genetics research. J. Biosci. 35, 119–126 (2010). 20413916 10.1007/s12038-010-0014-6

[b57] Davis R. H. Neurospora: Contributions of a Model Organism. (Oxford University Press, 2000).

[b58] Colot H. V. . A high-throughput gene knockout procedure for *Neurospora* reveals functions for multiple transcription factors. Proc. Natl. Acad. Sci. USA 103, 10352–10357 (2006). 16801547 10.1073/pnas.0601456103 PMC1482798

[b59] Langmead B., Trapnell C., Pop M. & Salzberg S. L. Ultrafast and memory-efficient alignment of short DNA sequences to the human genome. Genome Biol. 10, R25, doi: 10.1186/gb-2009-10-3-r25 (2009). 19261174 PMC2690996

[b60] Robinson M. D., McCarthy D. J. & Smyth G. K. edgeR: a Bioconductor package for differential expression analysis of digital gene expression data. Bioinformatics 26, 139–140, doi: 10.1093/bioinformatics/btp616 (2010). 19910308 PMC2796818

[b61] Benjamini Y. & Hochberg Y. Controlling the false discovery rate: a practical and powerful approach to multiple testing. J. Roy. Stat. Soc. Ser. B 57, 289–300 (1995).

