# Supplementary material for: A Ras GTPase associated protein is involved in the phototropic and circadian photobiology responses in fungi
Source: Sci Rep. 2017 Mar 21;7:44790. doi: 10.1038/srep44790 (PMC5359613; doi:10.1038/srep44790)
Supplement: Supplementary Material [file srep44790-s1.pdf]

SUPPLEMENTARY MATERIAL

A Ras GTPase associated protein is involved in the phototropic and circadian photobiology responses in fungi

Silvia Polaino[1], José M. Villalobos-Escobedo[2], Viplendra P.S. Shakya[1], Alejandro Miralles-Durán[3], Suman Chaudhary[1], Catalina Sanz[4], Mahdi Shahriari[4], Eva M. Luque[3], Arturo P. Eslava[4], Luis M. Corrochano[3], Alfredo Herrera-Estrella[2] & Alexander Idnurm[1,5]

[1]Division of Cell Biology and Biophysics, School of Biological Sciences, University of Missouri-Kansas City, Kansas City, USA. [2]Laboratorio Nacional de Genómica para la Biodiversidad, CINVESTAV Sede Irapuato, Irapuato, Guanajuato, Mexico. [3]Departamento de Genética, Universidad de Sevilla, Sevilla, Spain. [4]Departamento de Microbiología y Genética, Universidad de Salamanca, Salamanca, Spain. [5]School of BioSciences, University of Melbourne, Australia.
Correspondence and requests for materials should be addressed to A.I. (email: alexander.idnurm@unimelb.edu.au)

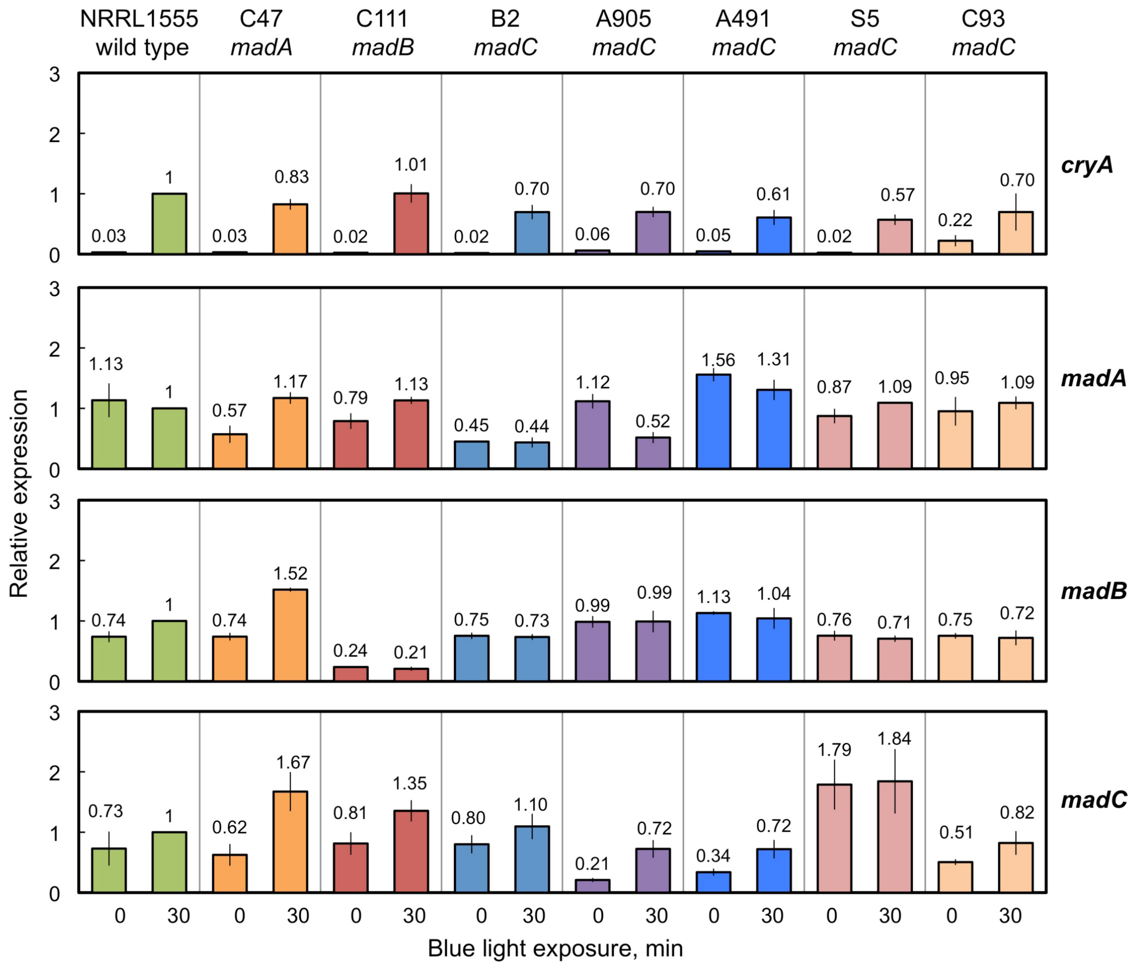

Figure S1. Regulation of transcript levels by blue light on the *P. blakesleeanus madA*, *madB*, *madC* and *cryA* genes. Total RNAs were isolated from mycelia exposed to 30 minutes of blue light, or kept in the dark. The amount of mRNA for each gene was assayed by quantitative RT-PCR. The data show the relative expression normalized to the wild type 30 min light culture, from three independent experiments.

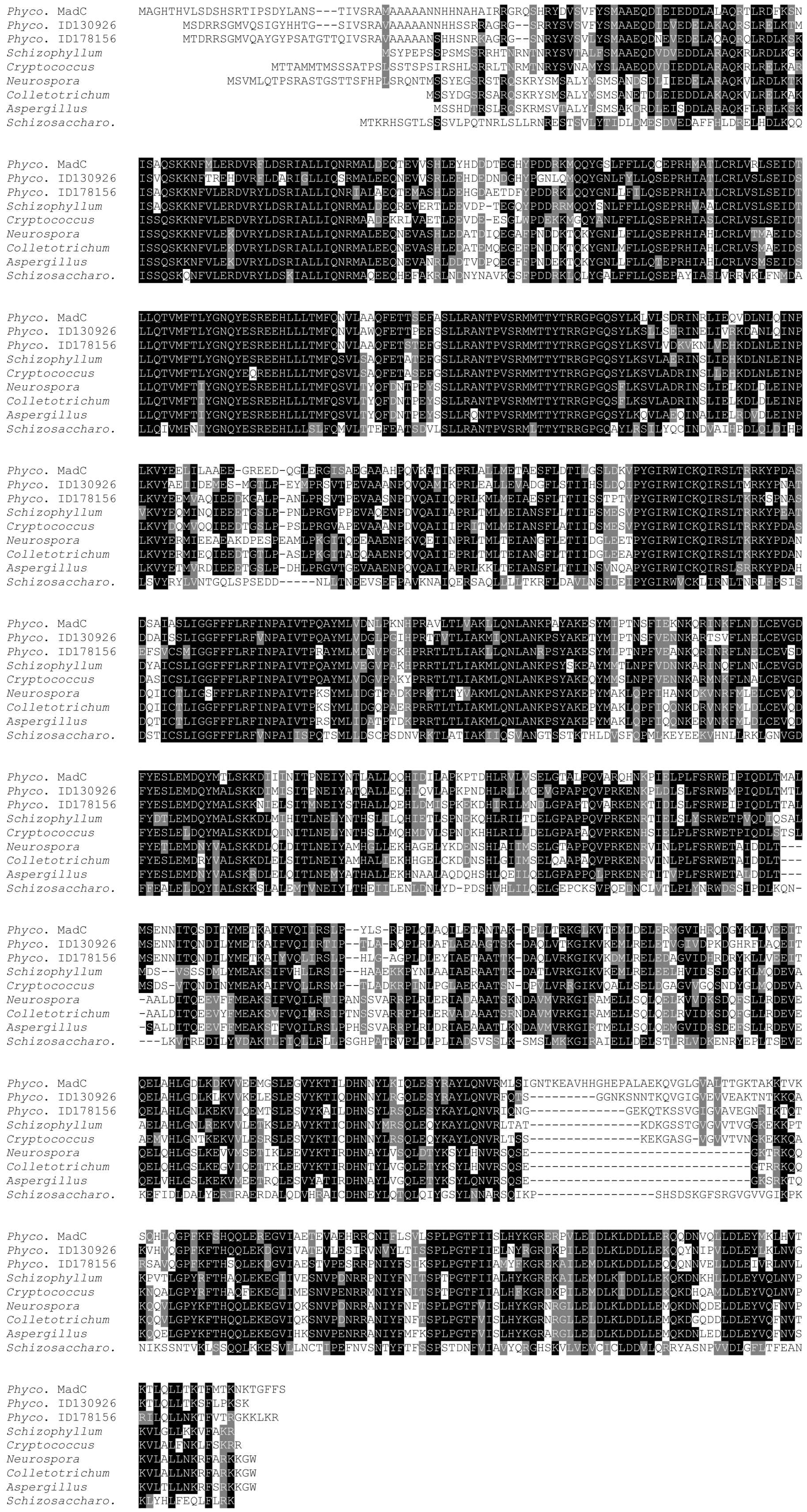
Phyco. MadC
Phyco. ID130926
Phyco. ID178156
Schizophyllum
Cryptococcus
Neurospora
Colletotrichum
Aspergillus
Schizosaccharo.

Figure S2. Alignment of the predicted protein sequence of MadC from *P. blakesleeanus* with homologs from other fungi. Two other homologs are present in *P. blakesleeanus* (*Phyco.*). The other homologs have been characterized in *Schizophyllum commune*, *Cryptococcus neoformans*, *Neurospora crassa*, *Aspergillus nidulans* and *Schizosaccharomyces pombe*, although for space and clearer resolution the Ira1 and Ira2 proteins from *Saccharomyces cerevisiae* are not included in the alignment.

Table S1. Strains used or created in this study (excluding the progeny from *P. blakesleeanus* crosses using in positional cloning). The *N. crassa ira-1*::*hph* mutants have been deposited to the Fungal Genetics Stock Center (FGSC).

Species/strain	Genotype	Origin
Phycomyces blakesleeanus		
NRRL1555	Wild type (–)	
NRRL1554	Wild type (+)	
UBC21	Wild type (+)	
UBC24	Wild type (–)	
A202	*madC469* (–)	A56 × B24
A914	*madC712, lysA401* (–)	A637 × A845
A491	*madC401* (–)	NRRL1555
A492	*madC402* (–)	NRRL1555
A905	*madC406* (–)	NRRL1555
B2	*madC452* (–)	NRRL1555
B3	*madC453* (–)	NRRL1555
B4	*madC454* (–)	NRRL1555
C39	*madC27* (+)	NRRL1554
C54	*madC42* (+)	NRRL1554
C93	*madC84* (+)	NRRL1554
C148	*carA5, madC119* (–)	C2
L1	*madC119* (–)	C264 × C148
L72	*madA7, madB103, madC119* (–)	L2 × L51
S5	*carA51, madC202* (–)	UBC24
S47	*carA86, madC232* (–)	UBC24
S193	*madC233* (–)	UBC21 × S5
S196	*madC202* (–)	UBC21 × S5
S205	*madC211* (–)	UBC21 × S23
Cryptococcus neoformans		
KN99α	Wild type, *MAT*α	[1]
KN99**a**	Wild type, *MAT***a**	[1]
AI81	*bwc1::NAT, MAT*α	[2]
AI89	*bwc1::NAT*, *MAT***a**	[2]
AI261	*ira1::NAT*, *MAT*α	KN99α
AI285	*ira1::NAT*, *MAT***a**	AI261 × KN99**a**
AI287	*bwc1::NAT*, *ira1::NAT*, *MAT***a**	AI89 × AI261
AI292	*ira1::NAT*, *IRA1-NEO*, *MAT*α	AI261

Saccharomyces cerevisiae		
W303	*MAT***a**/*MAT*α *leu2-3,112 trp1-1 can1-100 ura3-1 ade2-1 his3-11,15*	[3]
PJ69-4A	*MAT***a** *trp1-901 leu2-3,112 ur3-52 his3,200 gal80Δ LYS2::GAL1-HIS3 GAL2-ADE2 met2::GAL7-lacZ*	[4]
Neurospora crassa		
FGSC 4200	Wild type, *mat a*	[5]
FGSC 2489	74-OR23-1VA	[6]
FGSC 22824	NCU06122 (*ira-1::hph mat A*)	FGSC 9718
FGSC 22825	NCU06122 (*ira-1::hph mat a*)	FGSC 9718
FGSC 9718	*mus-51::bar+ mat a*	[7]
FGSC 1858	NCU08823 (*bd mat A*)	[8]
FGSC 10787	*ira-1::hph bd mat A*	FGSC 22825 × FGSC 1858
FGSC 10788	*ira-1::hph bd mat a*	FGSC 10787 × FGSC 4200
FGSC 10786	*ira-1::hph mat A*	FGSC 22825 × FGSC 1858
FGSC 10785	*ira-1::hph mat a*	FGSC 10786 × FGSC 4200

Table S2. Functional category enrichment analysis. A Gene Ontology (GO)-term enrichment analysis was performed with the hypergeometric distribution method. GO terms with FDR ≤ 0.2 were considered significantly enriched in each comparison.

GO term	Wild type %	*madC* %	Wild type FDR	*madC* FDR
lipid metabolic process	10	9	0.03251849	0.147265712
signal transducer activity	15	11	0.03251849	1
glycerolipid metabolic process	33	25	0.126260728	1
signal transduction	6	7	1	0.199257395
electron carrier activity	9	11	1	0.199257395
biosynthetic process	-6	-1	0.000277736	1
carbohydrate metabolic process	-7	-2	0.045320883	1
alanine metabolic process	-38	0	0.101037198	1
aspartate metabolic process	-38	0	0.101037198	1

Table S3. Primers used in this study. Mapping primers are as described previously [9]. Lowercase regions in primers SP98 and SP99 correspond to the flanks of the *IRA1* gene from *S. cerevisiae*. Restriction enzyme sites used for subcloning are underlined.

Name	Sequence (5´-3´)	Purpose
ALID0428	TCAACGCAGATTGTGAAAGG	Amplify and/or sequence *madC* gene
ALID0429	CCTAAATCAAACGCTAACTG	
ALID0430	GATGCTGGGATTTTACGG	
ALID0431	TTTATTACATGATACCAC	
ALID0434	TTACTGCTACTGCTACTG	
ALID0936	ATGGATCAGTACATGACC	
SP106	GTCTTGGTGAGTGAACTG	
ALID1440	AACCATGTTTCAGAATGTATTGG	3´ RACE over intron
ALID1493	CTCTCCTACGGGCCAATACACC	3´ RACE nested
ALID1620	CCGTAAAATCCCAGCATC	3´ RACE sequencing
ALID1599	TTAGAGCCATGGTCAAGTCTTGGATTGG	5´ RACE
ALID1598	CAGCAGCACATCGATATCC	5´ RACE nested
ALID1621	ATCTGTAGGTTCAAATCG	5´ RACE sequencing
ALID1628	ATCGAATTCATGGCTGGCCACACACATG	To clone cDNA of *madC* gene, directly using EcoRI
ALID1629	TCAGAATTCACTTAGGAAAAGAAACCAGTC	
ALID1734	CATGGAGGCCGAATTCATGTCAAAAGCATCTTTTCTC	Yeast two hybrid (Y2H), RasA ID 188551
ALID1735	GCAGGTCGACGGATCCCATTAACACACAGCAGCATCC	
ALID1829	AAGCAGTGGTATCAACGCAGAGTGGCCCATGTCAAAAGCATCTTTTCTC	
ALID1830	TCTAGAGGCCGAGGCGGCCGACATGCATTAACACACAGCAGCATCC	
ALID1736	CATGGAGGCCGAATTCATGTCTAAACCATCTTCATTTGTG	Y2H, RasB ID 177348
ALID1737	GCAGGTCGACGGATCCCATCAAGATGCAGCACTTG	
ALID1831	AAGCAGTGGTATCAACGCAGAGTGGCCCATGTCTAAACCATCTTCATTTGTG	
ALID1832	TCTAGAGGCCGAGGCGGCCGACATGCATCAAGATGCAGCACTTG	
SP98	catatgctgatgtggaggcagattcagttttcaattcgtgtcggtccatcCGTACGCTGCAGGTCGAC	Deletion of *S. cerevisiae IRA1*
SP99	cagatgattgttctagggttgataacctcctgaattcctttctagtcagcATCGATGAATTCGAGCTCG	
SP100	GGAATACTCTTTGACCAAGC	Confirming *IRA1* gene replacement in *S. cerevisiae*
SP101	CTTCACAACTGACAAGCCTC	
SP102	CTGGATTGATTCTCGTGTGC	
SP103	GCCAGTTGTGGATGATGAAC	
KanB	CTGCAGCGAGGAGCCGTAAT	
KanC	TGATTTTGATGACGAGCGTAAT	
ALID0491<br>ALID0492<br>ALID0493<br>ALID0494<br>ai006<br>ai290	ACAGCTAAGCCTCTCAAG<br>GCTTATGTGAGTCCTCCCGGACATGGTCATCATA<br>GC<br>CTCGTTTCTACATCTCTTCTGTCATACTGGTCATG<br>G<br>TCCTGGAACGACATACTG	Deletion of *C. neoformans IRA1* serotype A

	GAAGAGATGTAGAAACGAG<br>GGGAGGACTCACATAAGC	
ALID0516	AGTGTGATCCTCGGTTAC	*C. neoformans IRA1* replacement 5′ screening
ALID0517	ATCTCTGCCCATTCCAGC	*C. neoformans IRA1* replacement 3′ screening
ALID2150<br>ALID2151	ACTAGTAATCAACTCATCGTGCTAC<br>GAGCTCAAGTTACAATCAAGCTGGC	Complementation of *C. neoformans IRA1*
cryA-78F<br>cryA-147R	GATCCACCCTGGAGATGTCAA<br>CCGTTTCCGAAAGGGTGTAA	RT-PCR of *Phycomyces cryA*
madA-135F<br>madA-204R	AGTGAAGATGTGGTCGGCTACTC<br>TGTCAAGAACGTAACGGAAGATTG	RT-PCR of *Phycomyces madA*
wctA-392F<br>wctA-465R	GCCATTTCTTCAAAGGATGTTTCT<br>TGTGTCCATCATGCGAGTAGACT	RT-PCR of *Phycomyces madB*
ras-gap-260F<br>ras-gap-360R	TGGCCCTTGACGAACAAAC<br>TCCGTATTGTTGCATTTTTCGA	RT-PCR of *Phycomyces madC*
act-98F<br>act-165R	AGGCTGTCCTTTCCCTTTACG<br>GACACCATCACCGGAATGG	RT-PCR of *Phycomyces actin*

Supplemental references

1 Nielsen, K. *et al.* Sexual cycle of *Cryptococcus neoformans* var. *grubii* and virulence of congenic **a** and α isolates. *Infect. Immun.* **71**, 4831-4841 (2003).

2 Idnurm, A. & Heitman, J. Light controls growth and development via a conserved pathway in the fungal kingdom. *PLoS Biol.* **3**, 615-626 (2005).

3 Rothstein, R. J. One-step gene disruption in yeast. *Method. Enzymol.* **101**, 202-211 (1983).

4 James, P., Halladay, J. & Craig, E. A. Genomic libraries and a host strain designed for highly efficient two-hybrid selection in yeast. *Genetics* **144**, 1425-1436 (1996).

5 Käfer, E. & Fraser, M. Isolation and genetic analysis of nuclease halo (*nuh*) mutants of *Neurospora crassa*. *Mol. Gen. Genet.* **169**, 117-127 (1979).

6 Mylyk, O. M., Barry, E. G. & Galeazzi, D. R. New isogenic wild types in *N. crassa*. *Neurospora Newsl.* **21**, 24 (1974).

7 Colot, H. V. *et al.* A high-throughput gene knockout procedure for *Neurospora* reveals functions for multiple transcription factors. *Proc. Natl. Acad. Sci. USA* **103**, 10352-10357 (2006).

8 Sargent, M. L. & Woodward, D. O. Genetic determinants of circadian rhythmicity in *Neurospora*. *J. Bacteriol.* **97**, 861-866 (1969).

9 Chaudhary, S., Polaino, S., Shakya, V. P. S. & Idnurm, A. A new genetic map for the zygomycete fungus *Phycomyces blakesleeanus*. *PLoS One* **8**, e58931 (2013).
